# Predictors of *Helping Babies Breathe* knowledge and skills among nurses in primary health settings in Dodoma region, Tanzania

**DOI:** 10.1186/s12884-020-2782-9

**Published:** 2020-03-12

**Authors:** Angelina A. Joho, Stephen M. Kibusi, Ipyana Mwampagatwa

**Affiliations:** 1 Department of Nursing and Midwifery, College of Health Sciences, University of Dodoma, Dodoma, Tanzania; 2 Department of Public Health, College of Health Sciences, University of Dodoma, Dodoma, Tanzania; 3 Department of Clinical Medicine, College of Health Sciences, University of Dodoma, Dodoma, Tanzania

**Keywords:** Helping babies breathe, Neonatal, Asphyxia, Emergency, Nurses, Dodoma

## Abstract

**Background:**

Globally, birth asphyxia is one of the leading causes of neonatal death. In Tanzania, neonatal deaths are estimated to be 25 deaths per 1000 live births and birth asphyxia accounts for 31% of those deaths.

**Method:**

A cross-sectional study was conducted in 40 health centers within 7 districts in Dodoma Region among nurses working in maternity units. Simple random sampling was used to select participants. A knowledge questionnaire and performance skills checklist were used to assess nurses’ knowledge and skills respectively. Chi-square and binary logistic regression were employed to test association and identify significant predictors of HBB knowledge and skills.

**Results:**

A total of 172 participants completed the study out of 176 recruited. This represents a respondent rate of 98%. Findings indicate that age, duration of professional training, and experience in maternity were significant predictors for knowledge and skills. However, after control of the confounders, experience in the maternity unit was found to be the only significant predictor of knowledge and skills in resuscitation of the neonates (AOR = 2.94; CI: 0.96–8.98; *P* = 0.05) and (AOR = 4.14; CI: 1.12–15.31; *P* = 0.03) respectively. Nurses with longer maternity nursing care experience of 5 years and above were better able to answer questions that demonstrated adequate knowledge (53.9%) and perform skills correctly (53.2%) related to HBB. Those with less than 5 years’ experience had limited knowledge (20%) and skills (10.5%).

**Conclusion:**

In this setting, direct work experience in the maternity unit was the main factor influencing knowledge and skills in neonatal resuscitation with HBB**.**

## Background

Worldwide, neonatal deaths are a challenge and are estimated to be 18 per 1000 live births as of 2017. This represents a decrease of 51% from 1990 [1]. In Tanzania in 2016, neonatal deaths were estimated to be 25 deaths per 1000 live births. This represents only a decrease of 3.9% from 2010 [2]. In Tanzania three quarters of the neonatal deaths are due to three conditions. These include; birth asphyxia or failure to initiate spontaneous breathing (31%), preterm complications (25%) and sepsis (20%) [3, 18]. In this setting, approximately 80% of neonatal deaths are related to a low birth weight due to prematurity and small for gestational age [3, 4]. The first 24 h after birth are vital for neonatal survival as 60–70% of neonatal deaths occur in first 24 h of birth [4].

Neonates with hypoxic events during the birth process may develop a long term sequela including cerebral palsy, seizure disorder, motor disorder, learning problems, and development delays [5]. To help the prevention of neonatal deaths and birth asphyxia birth attendants need knowledge and skills in a basic neonatal resuscitation such as the Helping Babies Breathe program (HBB).

HBB uses a framework of the “golden minute” whereby the goal is for a baby to either be breathing or ventilated with a bag and mask within 1-min following delivery. The basic steps of HBB resuscitation are initiated at the time of delivery. The new-born is evaluated for crying and breathing. If the baby is crying strongly, it is breathing well and can continue with routine monitoring with its mother.

A baby who doesn’t cry will need to undergo the basic steps of resuscitation [6]. The sequence includes thoroughly drying the baby. This process of drying serves to stimulate the baby to transition and begin breathing. The second step involves keeping baby warm and clearing the airway (if needed) to remove visible secretions or meconium. Suction is minimized because it can cause bradycardia, apnoea or trauma. A bulb syringe or penguin suction device is used to quickly and gently suction the baby’s mouth and then each nostril. The third step in the process is to initiate effective ventilations with an appropriately sized bag and mask. With these simple steps most babies will transition and begin breathing on their own. Very few babies will need advanced resuscitation including specific medications and cardiac resuscitation [7, 20].

Despite a clear evidence of the efficacy of the HBB model, this intervention has been underutilized in many settings. Similar to other low resource settings, Tanzania continues to struggle with a shortage of well-trained staff and shortage of HBB equipment and providers with less than optimal resuscitation skills [8, 21].

Skilled birth attendants with intrapartum care knowledge and skills can handle intrapartum complications including prevention and management of birth asphyxia. These providers are able to identify, manage, and refer mothers and neonates with complications. A skilled birth attendant should be able to perform all signal functions of emergency maternal and newborn care, including HBB, to optimize newborn health and well-being [6].

In many cases, deaths due to intrapartum related complication (neonatal birth asphyxia) are a reflection of knowledge and skills of frontline health care provided to neonates in our setting. This study sought to assess factors influencing Helping Babies Breathe knowledge and skills among nurses in the primary health care setting in Dodoma Region, Tanzania.

## Methods

A descriptive cross-sectional study involved 172 nurses working in the health centers in all seven districts of the region. Dodoma Region is located in the central zone of Tanzania and was chosen for this study because of a high maternal death rate of 512 per 100,000 livebirths and high neonatal death rate of 32 per 1000 in 2015 [9]. Dodoma region had a total 69,843 deliveries in 2017 [6].

### Sampling size and sample technique

This study is the baseline from a quasi-experimental study. The sample size was calculated using the formula below [10].

$$ \mathrm{N}=\frac{{\left\{ Z\alpha \surd \left[\pi o\ \left(1-\pi o\right)\right]+2\beta \surd \left[\pi 1\left(1-\pi 1\right)\right]\right\}}^2}{\left(\pi 1-\pi o\right)2} $$

Where n = maximum sample size. Ζα = Standard normal deviation (1.96) at 95% confidence level for this study. 2β = standard normal deviate (0.8) with a power of demonstrating a statistically significant difference before and after the intervention between the two groups at 80%. πο = Proportion at pre- intervention (baseline skills on PPH management 39% [11]. π1_=_ proportion after intervention (proportion skills after intervention 50%) [11]. Study conducted at Haydom Lutheran Hospital in Northern of Tanzania.

$$ \mathrm{n}=\frac{{\left\{1.96\surd \left[0.39\left(1-0.39\right)\right]+0.8\surd \left[0.5\left(1-0.5\right)\right]\right\}}^2}{{\left(0.50-0.39\right)}^2} $$

***with adjustment for 15% attrition*** (Amico, 2009)***, the calculated sample size is 176.***

Therefore, the minimum sample size was 152 nurses. However, the researchers sampled 176 nurses to increase the power of the statistical tests and to plan for non-responses, which were expected to be less than 15%.

### Sample technique

All health centers in Dodoma Region were included in this study. Maternal units (labor and postnatal wards) and obstetric operating theaters in these health centers were purposively selected as nurses in the maternity units and obstetric surgical theaters work together and share responsibility of providing newborn resuscitation when necessary. Nurses from each these units were randomly selected. This study assessment was a baseline study performed prior to a formal HBB training. All nurses included in this study had not yet received on the job training in Helping Babies Breathe.

### Data collection procedure

Data collection occurred at the health centers where participants worked. The data collection was conducted by two master trainers of Helping Babies Breathe who are health sciences faculty at the University of Dodoma in Dodoma, Tanzania. Knowledge related to HBB was assessed using the standardized knowledge check questionnaire which comes with HBB training materials. This checklist was developed by the American Academic of Pediatrics (AAP) in 2009 (version 1) and has been utilized in various research projects in Tanzania and beyond. It has been updated in recent years with the evolution of the HBB training materials [10, 11]. The questionnaire contained 17 multiple choice questions. Each item with a correct response scored one mark and zero for an incorrect response. A respondent who answered at least 14 out of 17 correctly was considered, to have adequate knowledge [10–12].

Helping Babies Breathe performance skills were assessed by using a standard observational checklist that is also included in the HBB training materials developed by AAP. The methodology includes evaluation of a participant’s skills using an Objective Structured Clinical Evaluation (OSCE). OSCE B from the HBB training materials (Version 1) was utilized for this study.

OSCE B is a comprehensive resuscitation skill testing and participants were expected to correctly perform four key steps of newborn resuscitation. This includes the following: 1) recognize baby is not breathing; 2) ventilate at 40 breaths per minute; 3) look for appropriate chest movement; and 4) perform the five steps to improve ventilation (reposition head, reapply mask, clear secretions, opens mouth slightly, squeeze bag harder). Successful completion of OSCE B requires a total score of 14 correct of 18 [10, 11, 19].

### Data analysis

Data were entered, cleaned and analyzed by using Statistical Package for Social Sciences (SPSS) version 20.0. Descriptive analysis was performed to determine the distribution of the background characteristics of the respondents. Thereafter, a chi-square test was performed to identify the variables associated with the nurses’ knowledge and skills related to HBB. Statistical significance was set at *P* < 0.05. Variables which showed statistically significant (P < 0.05) were further modeled to multiple logistic regression (both reduced and full model) to identify the significant predictors of nurses’ knowledge and skills on HBB.

## Results

### Socio-demographic characteristics of respondents

In the current study we recruited 176 nurses and 172 participants completed the study. This represents a respondent rate of 98%. The majority of respondents were 40 years or older (40.7%), and 27 (15.7%) were male and 145 (84.3%) were female. Regarding work experience; 77 (44.8%) had maternity work experience of more than 5 years and 95 (55.2%) had less than 5 years of experience. In terms of level of professional, 109 (63.4%) were enrolled nurses and only 63 (36.6%) were registered nurses (Table 1).

**Table 1:** Distribution of Nurses by Demographic Characteristics

Demographic characteristics		Frequency	Percent
Sex	Male	27	15.7
	Female	145	84.3
Age Group (Years)	20–29	63	36.6
	30–39	39	22.7
	40 and above	70	40.7
Level of professional	Enrolled nurses	109	63.4
	Registered nurses	63	36.6
Years of professional training	Less than or equal to 3	120	69.8
	Above 3	52	30.2
Experience in maternity	Less than 5 years	95	55.2
	Above 5 years	77	44.8

### Knowledge and skills regarding HBB

Based on operational definition of this study, participants completed the knowledge check which included 17 multiple choice questions, each equally weighted. A score of 14 or above was considered as a passing grade. Among our participants only 40.1% scored 14 or above. The majority of the participants (59.9%) did not have adequate knowledge in Helping Babies Breathe.

The same participants then participated in a structured evaluation of their skills by using OSCE B. This OSCE contains 18 items and a score of 14 and above was required to demonstrate adequate skills in HBB [10]. Among the assessed nurses only 29.7% performed Helping Babies Breathe skills correctly while 70.3% had inadequate skills in HBB (Table 2).

**Table 2:** Distribution of nurse’s knowledge and skills of HBB

Item	Correct	Incorrect
Knowledge	41.1	59.1
Skills	29.7	70.3

### Factors influencing knowledge and skills related to HBB

Factors influencing knowledge and skills related to HBB were explored by using a chi-square test and binary logistic regression. The chi-square test explored the association between socio-demographic variables and knowledge and skills of HBB. Then binary logistic regression models were utilized to identify significant predictors of knowledge and skills of HBB.

#### Association between nurses’ background characteristics, with knowledge and skills related to HBB

Among our respondents 19% of the nurses within age category 20 to 29 years, 51.3% between the ages of 30 to 39 years and 52.9% older than 40 years -- were able to answer questions correctly and demonstrate adequate HBB knowledge (*P* = 0.01; *x*^2^_=_ 18.39). Nurses with 3 years of professional training were better able to answer correctly (59.6%), while only 31.7% of nurses with less than 3 years were found to have adequate knowledge (*P* = 0.01; *x*^2^_=_ 11.80). Among respondents with longer work experience in maternity units, 53.9% had adequate knowledge. This represents a significant association with knowledge of HBB at the 5% level of significance (*P* = 0.01; *x*^2^_=_ 19.88).

In terms of skills related to HBB, 33.1% of female nurses were able to perform skills correctly, while only 11.1% of male nurses performed correctly (*P* = 0.022; *x*2 = 5.28). For the age category, 9.4% of providers between the ages of 20 to 29 years demonstrated adequate skills, 25.6% of providers between ages 30 to 39 demonstrated adequate skills and 50.7% of nurses above 40 years performed the HBB skills correctly (*P* = 0.01; *x*2 = 27.61). Duration of professional training appeared to impact skill performance as 53.8% of participants with more than 3 years of nursing education were able to perform skills correctly, while only 19.2% with less than 3 years of education performed skills correctly (*P* = 0.01; *x*2 = 20.92).

Among nurses with at least 5 years of work experience, 53.2% performed skills correctly. In comparison, only 10.5% of nurses with less than 5 years of work experience performed skills correctly (*P* = 0.001; *x*2 37.21). Among nurses who worked during the night shift, 47.3% performed skills correctly, while among nurses working during the day only 21.4% were able to perform skills correctly (*P* = 0.01; *x*212.04)_._

In summary, three background characteristics were found to have a significant association with resuscitation knowledge and five background characteristics had a significant association with skills of HBB. Age, duration of professional qualification and maternity unit work experience had a significant association with both knowledge and skills (*P* < 0.01) (Table 3).

**Table 3:** Results of Chi-square analysis of Nurses Knowledge and skills of HBB versus the Background Characteristics (*N* = 172)

Demographic characteristics		HBB Knowledge		*x*^2^	Sig.	HBB Skills		*x*^2^	Sig
		Incorrect response (%)	Correct response (%)			Inadequate performance (%)	Adequate performance (%)		
Sex	Male	19(70.4)	8 (29.6)	1.47	0.23	24 (88.9)	3 (11.1)		0.02
	Female	84 (57.9)	61 (42.1)			97 (66.9)	48 (33.1)	5.28	
Age Group (Years)	20–29	51 (81.0)	12 (19.0)	18.39	0.00	58 (90.6)	6 (9.4)		0.00
	30–39	19 (48.7)	20 (51.3)			29 (74.4)	10 (25.6)		
	≥ 40	33 (47.1)	37 (52.9)			34 (49.3)	35 (50.7)	27.61	
Professional qualification	EN	70 (64.2)	39 (35.8)	2.33	0.13	78 (70.9)	32 (29.1)		0.83
	RN	33 (52.4)	30 (47.6)			43 (69.4)	19 (30.6)	0.05	
Duration of Professional training	≤ 3 years	82 (68.3)	38 (31.7)	11.80	0.00	97 (80.0)	23 (19.2)		0.00
	> 3 years	21 (40.4)	31 (59.6)			24 (46.2)	28 (53.8)	20.92	
Experience in maternity	< 5 years	56 (80.0)	14 (20.0)		0.00	85 (89.5)	10 (10.5)		0.00
	≥ 5 years	47 (46.1)	55 (53.9)	19.88		36 (46.8)	41 (53.2)	37.21	
Night shift	No	15 (55.6)	12 (44.4)			92 (78.6)	25 (21.4)		0.00
	Yes	88 (60.7)	57 (39.3)	0.25	0.62	29 (52.7)	26 (47.3)	12.04	

#### Identification of significant predictors of knowledge and skills related to HBB

Logistic regression was performed to identify the background characteristics of nurses, which were significant predictors of knowledge and skills related to HBB at the 5% level of significance.

#### Significant predictors of knowledge related to HBB

Unadjusted associations with knowledge related to HBB indicated that nurses with more than 3 years professional training were associated with increased likelihood of adequate knowledge compared to those with less than 3 years of professional training (OR = 3.19; CI: 1.62–6.25: *P* < 0.05).

Longer work experience (more than 5 years) was associated with increased likelihood of adequate HBB knowledge compared to nurses with less than 5 years of work experience in maternity units. (OR = 4.16; CI: 2.18–7.95: *P* < 0.05).

After adjusting all variables (full model), only years of work experience in the maternity unit was found to be a significant predictor. The adjusted odds ratio showed that nurses with longer working experience (more than 5 years) in the maternity unit was associated with increased likelihood of adequate HBB knowledge (AOR = 3.28; CI:1.52–7.08: *P* < 0.05) (Table 4).

**Table 4:** Binary Logistic Regression for unadjusted (crude) and adjusted Odds Ratio, for factors associated with knowledge on HBB among Nurses in Dodoma (*n* = 172)

Background characteristics	OR	*P* value	95% C.I.		AOR	*P* value	95% C.I.	
			Lower	Upper			Lower	Upper
Duration of professional training								
≤ 3 years	1							
> 3 years	3.19	0.00	1.62	6.25	1.58	0.27	0.70	3.59
Experience in maternity								
< 5 years	1							
≥ 5 years	4.16	0.00	2.18	7.95	3.28	0.00	1.52	7.08

#### Significant predictors of skills related to HBB

Unadjusted associations of HBB skills indicated that sex, age of 40 and above, years of professional training, and experience in maternity unit and night shifts were all significant predictors of HBB skills. Female nurses were associated with the increased likelihood of correct HBB skills performance compared to male nurses (OR = 3.96; CI: 1.14–13.81). Nurses 40 years and older were associated with an increased likelihood of performing HBB correctly compared to nurses between 20 and 29 years old (OR = 9.50; CI: 3.63–24.88). Those with more than 3 years of professional training had an increased likelihood of correct HBB skills compared to nurses with less than 3 years of professional training (OR = 4.92; CI: 2.42–10.01). Longer working experience (more than 5 years in the maternity unit) was associated with an increased likelihood of correct skills in HBB (OR = 9.68; CI: 4.38–21.41). Nurses who worked during the night shift were more likely to be skilled compared to those on the day shift (OR = 0.32; CI: 0.16–0.63).

After controlling other variables (full model), only longer work experience in maternity was associated with an increased likelihood of performing HBB skills correctly. Direct work experience on the maternity unit was revealed to be a significant predictor. Its adjusted odds ratio demonstrated that nurses who had five or more years’ experience in maternity were more than four times likely to be skilled (AOR = 4.14; CI: 1.12–15.31) (Table 5).

**Table 5:** Binary logistic regression for unadjusted (crude) and adjusted odds ratio, for factors associated with skills of HBB among nurses in Dodoma Region (n = 172)

Demographic characteristics	OR	*P* value	95% C.I.		AOR	*P* value	95% C.I.	
			Lower	Upper			Lower	Upper
Sex								
Male	1							
Female	3.96	0.03	1.14	13.81	1.65	0.48	0.41	6.71
Age (years)								
20–29	1							
30–39	3.28	0.04	1.08	9.91	0.53	0.56	0.06	4.51
40 and above	9.50	0.00	3.63	24.88	0.71	0.76	0.08	6.48
Duration of professional training								
less than 3 years	1							
3 years and above	4.92	0.00	2.42	10.01	1.39	0.47	0.57	3.41
Experience in the maternity unit								
less than 5 years	1							
5 years and above	9.68	0.00	4.38	21.41	4.14	0.03	1.12	15.31
Current working in night shift								
No	1							
Yes	0.32	0.00	0.16	0.63	0.42	0.03	0.19	0.93

## Discussion

This baseline study aimed to assess Helping Babies Breathe knowledge and skills among frontline nurses *and* to identify influencing factors in this setting. A large discrepancy between knowledge and skills was observed. While 59.9% of nurses passed the knowledge questions and demonstrated adequate knowledge, only 29.7% demonstrated adequate skills in newborn resuscitation using the basic steps of Helping Babies Breathe. More than half of nurses (57%) did not begin ventilations within 1 min and were unable to ventilate at 40 breaths per minute.

Moreover, half of the nurses (50%) ventilated babies ineffectively by failing to open the airway (reposition the head, reapply mask and cleans secretions). Their performance may be reflective of inexperience in using a bag and mask or lack of exposure to OSCE based evaluation. However, their performance may be reflective of steps taken during resuscitation of real neonates. Our findings are consistent with previous studies that reported low scores in HBB knowledge and skills among birth attendants [11] and the worrisome gap between HBB knowledge and skills performance [8].

Low knowledge and skills in neonatal resuscitation observed in the current study are of grave concern as they may reflect low knowledge and skills in other areas of maternal and neonatal care. Appropriate knowledge and skills in management of maternal and neonatal emergencies is critical to avoid delays in life saving care at the health facility level [13]. System gaps such as lack of resuscitation equipment and supplies also influences delays at the health facility [14]. Better understanding of the factors influencing delays would help the health care system to improve the quality of care and corresponding outcomes. One third of neonatal deaths are among neonates needing HBB, but too often frontline workers attending birth do not have adequate knowledge or skills in basic newborn resuscitation [15]. This huge gap in skills could be easily remedied. There is a clear need for health professional educators to integrate knowledge and skills in basic newborn resuscitation when training pre-service and in-service health professionals. By exposing health professional students to HBB, it may better ensure that they have the requisite knowledge and skills to safely attend birth and resuscitate neonates as students completing clinical rotations on the wards and in the future as in-service professionals.

Working experience of more than 5 years was a significant predictor of a nurse’s knowledge and skills related to HBB [22]. Knowledge can be obtained through reading and lectures. However, skill development can occur through a variety of approaches. Among our sample, the nurses likely gained experienced through their work on the maternity wards through observation and mentorship over time. This demonstrates that it is possible to gain competence in HBB knowledge and skills through work exposure as none of our participants had received prior training in HBB. However, in order to reduce preventable neonatal deaths, there is a need for our local education and health systems to adopt innovative strategies to accelerate mastery of knowledge and skills in newborn resuscitation among health professional students and in-service providers. Prevention of birth asphyxia, remains a priority for reducing neonatal mortality. Our study demonstrates that frontline nurses attending birth in Dodoma region would likely benefit from training in HBB followed by timely refresher trainings and mentorship to reduce neonatal deaths [16].

Various studies have reported that increased years of working experience in the maternity unit resulted in improved neonatal resuscitation skills and self-efficacy, leading to improved neonatal outcomes [12, 17]. It is however reported that, nurses who will be having experiences in maternity units were expected to pass knowledge and skills to others including the newly employed in the neonatal resuscitation [12].

This study is not without limitations. The assessment of nurses’ HBB knowledge and skills demonstrated in this evaluation, may not translate into their actual practice. The evaluation of actual resuscitations in the clinical environment using a standardized checklist may better demonstrate the actual performance of nurses. Evaluating nurses performing newborn resuscitation during deliveries before and after HBB training might be a preferred approach. Additionally, in this study we did not achieve the calculated minimum sample size because four nurses declined to participate in the study. This study was only conducted only at seven primary health care facilities. For this reason, our findings cannot not be generalized to the whole region.

## Conclusion

This study has revealed low knowledge and skills on HBB among nurses working in the primary health settings in Dodoma Region, Tanzania. The predictor observed was work experience in the maternity unit for more than 5 years. To reduce neonatal deaths from birth asphyxia there is an urgent need for effective interventions to accelerate the knowledge and skills of nurses in neonatal resuscitation.

## Data Availability

Data set is available upon request to the corresponding author.

## Abbreviations

AAP: American Academy of Pediatrics
HBB: Helping Babies Breathe
NICHP: National Institute of Child Health and Human Development
NRP: Neonatal Resuscitation
OSCE: Objective Structured Clinical Examination
TDHS: Tanzanian Demographic and Health Survey
WHO: Word Health Organization
